# Volumetric absorptive microsampling for profiling of signaling lipids: a comparative analysis with whole blood and dried blood spots

**DOI:** 10.1007/s00216-026-06413-5

**Published:** 2026-04-05

**Authors:** Manchu Umarani Thangavelu, Alida Kindt, Bert Wouters, Lieke Lamont, Hyung Lim Elfrink, Amy Harms, Thomas Hankemeier

**Affiliations:** Metabolomics and Analytics Center, Leiden University, Einsteinweg 55, 2333 CC Leiden, The Netherlands

**Keywords:** Volumetric absorptive microsampling, Dried blood spot, Signaling lipids, Pre-analytical variability, Stability

## Abstract

Signaling lipids regulate key physiological and pathological processes, providing insights into disease mechanisms and therapeutic targets. While plasma is the standard biofluid for their analysis, its collection and handling pose challenges, including the need for venous access, immediate processing, and stringent cold-chain transportation to prevent lipid degradation. Volumetric absorptive microsampling (VAMS) has emerged as an alternative to conventional whole blood (WB) and dried blood spot (DBS), yet its suitability for comprehensive signaling lipid profiling, including low-abundance species, remains unexplored. This study evaluated the feasibility of VAMS for profiling diverse signaling lipid classes using a single liquid-liquid extraction (LLE) protocol. Analytical performance was assessed by evaluating precision, LLE recovery, matrix effects, and endogenous metabolite levels across WB, DBS, and VAMS. Short-term stability of VAMS at room temperature was examined over 24h, 48h, and 1 week storage. VAMS demonstrated superior precision compared to DBS while achieving moderate but comparable recoveries. The metabolic profile of VAMS aligned closely to WB, while DBS deviated substantially, demonstrating improved lipid stability in VAMS in the initial 24h period at room temperature. However, extended storage for 1 week introduced significant artifacts, altering metabolome composition and emphasizing the need for stabilization strategies, such as desiccant-assisted storage or antioxidant pretreatment to minimize hydrolytic and oxidative degradation. These findings provide initial evidence supporting VAMS as a feasible and promising alternative to DBS for signaling lipid profiling. Further studies are needed to assess extraction efficiency from the VAMS tip, optimize LLE recovery, and evaluate long-term stability, advancing the applicability of VAMS in lipidomics research.

**Graphical abstract:**

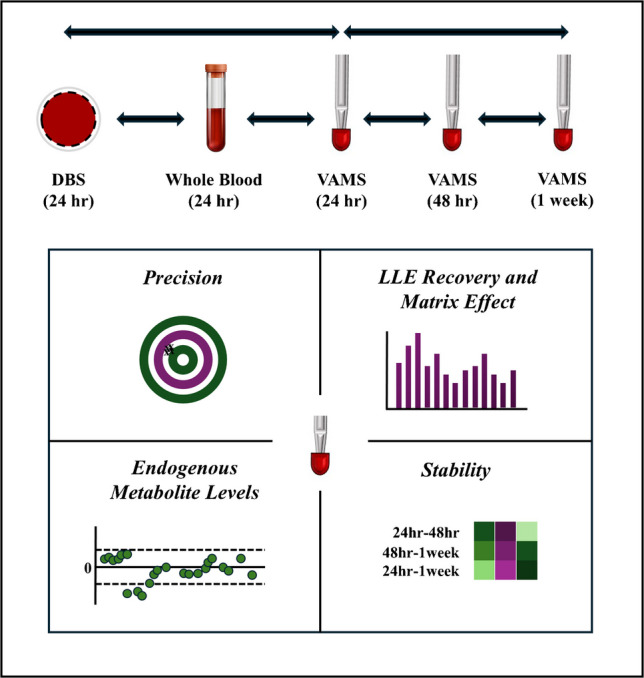

**Supplementary Information:**

The online version contains supplementary material available at 10.1007/s00216-026-06413-5.

## Introduction

Lipidomics is the large-scale study of lipids within biological systems, encompassing their identification, characterization, and differentiation [1]. Dyslipidemia, defined as an imbalance in lipid composition in the body, has been implicated in the pathogenesis of various diseases, including degenerative disorders and COVID-19 [2–4]. While structural lipids are primarily involved in membrane architecture and energy storage, a distinct subset of lipids functions as bioactive signaling molecules, modulating key physiological and pathological processes [5–8]. Signaling lipids are dynamic messengers that govern cellular communication, inflammation, immune response, vascular tone, and tissue remodeling, serving as essential regulators of homeostasis [6, 9, 10]. The transient nature of their biosynthesis and degradation facilitates a precise, dynamic, and context-dependent modulation of cellular responses dictated by cellular state, tissue type, and stimuli [11, 12]. This intrinsic sensitivity underscores their potential as biomarkers for disease prediction, diagnosis, or prognosis, as well as strategic targets for therapeutic interventions. Dysregulated lipid signaling has been implicated in the pathogenesis of a broad spectrum of pathological conditions, including metabolic disorders, cardiovascular diseases, neurodegenerative conditions, and inflammatory syndromes [13–16]. The increasing recognition of their pivotal roles in health and diseases has driven a surge in signaling lipid research, leading to the discovery of novel lipid mediators, elucidation of complex metabolic pathways, and transition towards precision medicine [17–19]. Advancements in high-throughput analytical techniques, such as liquid-chromatography mass-spectrometry (LC-MS), have revolutionized lipidomics by enabling comprehensive lipid profiling, precise quantification, and in-depth characterization of signaling pathways [20]. However, the accurate measurement of signaling lipids poses inherent challenges due to their low abundance, rapid turnover, and susceptibility to pre-analytical variability, necessitating optimized sample collection and preparation strategies [21].

Recent research in our laboratory has led to the development of a rapid and comprehensive ultra-high performance liquid-chromatography tandem mass-spectrometry (UHPLC-MS/MS) method for targeted signaling lipid profiling in plasma [22]. The method has been optimized from Schoeman et al. with the inclusion of endocannabinoids and bile acids [23]. An MTBE-based extraction protocol allowed for the validation and quantification of diverse lipid classes, including oxylipins, lysophospholipids, free fatty acids, bile acids, endocannabinoids, and a steroid hormone. While plasma remains a widely used biofluid for lipidomic analyses, its collection and processing pose technical and logistical challenges, such as the need for trained personnel to perform venipuncture, centrifugation to isolate cellular components, and stringent cold-chain transport and storage to preserve sample stability [21]. A viable alternative to conventional plasma sampling is blood microsampling, a minimally invasive technique that enables the collection of small volumes of whole blood, typically < 100 µL, through a capillary puncture [24]. This approach eliminates the need for venous access, reduces sample handling complexities and biohazard risks, and facilitates remote or at-home self-sampling, making it particularly valuable for longitudinal studies and vulnerable patient populations [24]. The oldest and most widely implemented microsampling technique is the dried blood spot (DBS) sampling, where blood droplets are collected onto filter paper and allowed to dry under ambient conditions. While DBS offers numerous advantages, its performance is inherently constrained by the hematocrit effect, wherein variations in erythrocyte concentrations influence blood viscosity and spread dynamics on the filter matrix. This variability can induce uneven analyte distribution and volumetric inconsistencies, ultimately compromising the accuracy and reproducibility of lipid quantification [25]. Despite these limitations, DBS has been applied for signaling lipid analyses. Several studies have developed targeted methods for oxylipin profiling from DBS, enabling the measurement of a broad range of oxylipins with high precision, linearity, and recovery [26, 27]. Similarly, free fatty acids and lysophospholipids have been reported in targeted and untargeted DBS lipidomics workflows, with studies demonstrating variable stability across different storage conditions and lipid species [25, 28]. However, existing studies employ distinct analytical methods tailored to individual lipid classes rather than a unified comprehensive approach [26, 29, 30]. Several other microsampling approaches have been explored for lipid analysis beyond DBS. Plasma separation cards, for example, facilitate the passive isolation of plasma from whole blood, thereby minimizing cellular interferences. However, plasma yields remain limited and lipid recoveries were lower and variable compared to traditional plasma analysis [28]. Alternative approaches, such as capillary microsampling and dried plasma spots, provide improved volumetric accuracy and enhanced control over sample quality, yet they demand careful handling and remain insufficiently standardized for broad application in lipidomics [31].

Volumetric absorptive microsampling (VAMS) technology offers a promising alternative by enabling collection of fixed volumes of whole blood, independent of the hematocrit effect [32]. VAMS utilizes an absorptive polymeric tip mounted on a plastic handler that facilitates controlled and reproducible sample collection, thereby minimizing hematocrit-dependent variability, heterogenous sample distribution, and imprecise volumetric collection [32]. VAMS has been gaining significant traction in the field of pharmacokinetics and therapeutic drug monitoring, with recent studies focusing on the development and validation of assays for drugs [33]. The application of VAMS for metabolomic analyses remains mainly focused on amino acids and organic acids [34]. While a few studies have explored its potential in lipidomics, including untargeted lipidomic profiling and multi-omics approaches for assessing lifestyle-associated health changes, the reported findings are largely restricted to lipid species such as ceramides, diacylglycerols, triglycerides, phospholipids, lysophospholipids, and free fatty acids [35, 36]. Notably, in an untargeted study by Marasca et al. VAMS enabled the detection of a broader range of lipid species compared to DBS, highlighting its potential superior analytical performance for lipidomics applications [35]. However, an evaluation of the suitability of VAMS across a broader range of signaling lipids remains unexplored.

To address these limitations, the feasibility of VAMS for the profiling of oxylipins, lysophospholipids, free fatty acids, bile acids, endocannabinoids, and steroid hormones was evaluated by adapting our previously established comprehensive plasma-based method for whole blood analysis. In this study, we systematically investigate the analytical performance of VAMS in comparison to DBS and liquid whole blood by assessing (i) precision, liquid-liquid extraction (LLE) recovery, and matrix effect across all three matrices; (ii) endogenous metabolite levels to determine the degree of concordance between the sampling techniques; and (iii) stability of VAMS to examine the impact of storage durations (24h, 48h, and 1 week) at room temperature. This explorative study aims to provide insights into the applicability of VAMS for comprehensive signaling analyses and inform potential optimization strategies to enhance analytical performance.

## Methods

### Sample collection and storage

Venous blood was drawn from a healthy non-fasting volunteer into a K2 EDTA tube via venipuncture and processed for microsampling within 30 min of collection. Written informed consent was obtained in accordance with institutional ethical guidelines, and the sample was anonymized prior to analysis, with no personal identifiers available to the investigators. DBS samples were prepared by pipetting 30 µL of the EDTA-treated blood onto pre-labeled Whatman 903 protein saver cards and stored at room temperature for 24h to ensure complete drying. VAMS samples were collected using a 30-µL Mitra device obtained from Trajan Scientific and Medical, where the blood was absorbed onto the polymeric tip until full saturation, then left to dry and stored at room temperature for 24h (VAMS24), 48h (VAMS48), or 1 week (VAMS1w). The remaining blood was stored at 4 °C for 24h prior to aliquoting (30 µL) to generate liquid whole blood samples for analysis, hereafter referred to as whole blood (WB) samples. Each sample type was prepared in five replicates to ensure analytical reproducibility.

### Sample preparation

**Fig. 1:**
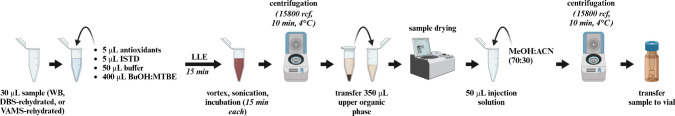
Schematic representation of sample preparation workflow for the analysis of signaling lipids. The figure illustrates key processing steps, including sample rehydration, addition of antioxidants and internal standards, and liquid-liquid extraction using BuOH:MTBE (1:1, v/v), to extract lipids from WB, DBS, and VAMS. The sample undergoes sequential processing through vortexing, sonication, and incubation before the upper organic phase is isolated, dried under vacuum, and reconstituted in MeOH:ACN (70:30, v/v) for subsequent LC-MS/MS analysis. ACN, acetonitrile; BuOH, butanol; DBS, dried blood spot; ISTD, internal standard; LC-MS/MS, liquid chromatography-tandem mass spectrometry; MeOH, methanol; MTBE, methyl tert-butyl ether; VAMS, volumetric absorptive microsampling; WB, whole blood

Entire DBS spots were excised from the filter cards, VAMS tips were detached from their plastic handles, and WB samples were aliquoted. Each sample was transferred into separate Eppendorf tubes for subsequent lipid extraction. The sample preparation protocol was adapted from our previously published plasma extraction methodology, with modifications to optimize the processing of DBS, VAMS, and WB samples. These adaptations included additional steps such as rehydration, vortexing, sonication, and incubation to ensure efficient analyte extraction across sample matrices. A detailed description of the chemicals, reagents, and protocol used in this study can be found in the work published by Yang et al. [22]. Briefly, the DBS and VAMS samples were rehydrated with 30 µL of Milli-Q water. All samples were spiked with 5 µL of antioxidant solution (0.2 mg/mL BHT and 0.2 mg/mL EDTA), 5 µL of internal standard (ISTD) solution composed of deuterated targets in 0.4 mg/mL BHT in MeOH, and 50 µL of 0.2 M citric acid and 0.4 M disodium hydrogen phosphate buffer (pH 4.5). Lipid extraction was performed via LLE using 400 µL of BuOH:MTBE (1:1 v/v), with samples left to equilibrate for 15 min. This was followed by a sequential processing consisting of vortexing, sonication, and incubation at room temperature, each for a period of 15 min, prior to centrifugation at 15,800 rcf for 10 min at 4 °C. A volume of 350 µL of the upper organic phase was separated, dried under vacuum using a speedVac, and reconstituted in a 70:30 (v/v) mixture of MeOH:ACN for subsequent analysis via LC-MS/MS (Fig. 1).

In addition to study samples, blank samples were prepared for each sample type using Milli-Q water as a surrogate matrix and processed using the same workflow, excluding the addition of ISTD solution. Similarly, post-spiked study samples for recovery and matrix effect calculation were prepared identical to the study samples, except that ISTDs were spiked post-LLE after the upper organic phase was dried, followed by a second drying step under vacuum before reconstitution. Neat samples were prepared by adding ISTD to an Eppendorf tube and dried under vacuum prior to reconstitution in a 70:30 (v/v) mixture of MeOH:ACN.

### Data acquisition by LC-MS/MS

Two distinct chromatographic methods were employed in accordance with the protocol established by Yang et al. [22]. Briefly, a low pH method was implemented for the analysis of oxylipins, endocannabinoids, and bile acids, while a high pH method was utilized for the separation of free fatty acids and lysophospholipids, spanning chain lengths from C14 to C22. Chromatographic separation was conducted using a Shimadzu Nexera X2, equipped with three high-pressure pumps (LC-30AD), a communication module (CBM-20Alite), an autosampler (SIL-30AC), and an oven (CTO-30A), supplied by Shimadzu Benelux. The low pH method employed a Waters BEH C18 column (2.1 × 50 mm, 1.7 µm), whereas the high pH method utilized a Kinetex EVO C18 column (2.1 × 50 mm, 1.7 μm). Mass spectrometric detection was conducted on a Sciex QTRAP 6500 system using electrospray ionization in polarity-switching mode with dynamic multiple reaction monitoring. Peak integration of the acquired data was performed on the vendor software Sciex OS (v2.1.6.59781).

### Data preprocessing and analysis

All descriptive statistics, statistical tests, and visualization were performed in RStudio (v4.3.1). To account for variability in sample processing and instrument performance, peak area ratios were calculated using the closest eluting ISTD for each metabolite. Outliers were identified through visual inspection using principal component analysis (PCA). The background interference was expressed as the percentage of signal intensity in blank samples to the signal intensity in study samples, computed using the equation:

$$\text{Background Signal }(\mathrm{\%}) =\frac{\text{Mean peak area of blank replicates}}{\text{Median peak area of study sample replicates}}\times 100$$

Precision was assessed using peak areas for ISTDs and area ratios for metabolites, expressed as the coefficient of variation (CV) using the equation:

$$\text{Precision }(\mathrm{\%})=\frac{\text{Standard deviation of study sample replicates}}{\text{Mean of study sample replicates}}\times 100$$

Quality control was performed to ensure data reliability, with metabolites reported only if background signal was below 40% and precision under 30%. LLE recovery and matrix effect were determined to assess method performance using the following equations:

$$\text{LLE Recovery }(\mathrm{\%})=\frac{\text{Mean of ISTD area in study sample replicates spiked pre LLE}}{\text{Mean of ISTD area in study sample replicates spiked post LLE}}\times 100$$

$$\text{Matrix Effect }(\mathrm{\%})=\frac{\text{Mean of ISTD area in study sample replicates spiked post LLE}}{\text{Mean of ISTD area in neat sample replicates}}\times 100$$

UpSet plots were used to illustrate the number of metabolites reported across the three matrices and their overlap between the sample types. All metabolite values were log_2_ transformed prior to statistical analysis. Pairwise comparisons between WB, DBS, and VAMS were conducted using *t*-tests and visualized through modified Bland-Altman plots and boxplots. Similarly, metabolite stability at room temperature across different storage durations (24h, 48h, and 1 week) in VAMS samples was assessed using *t*-tests and ANOVA. The results were visualized using a PCA plot and heatmaps to examine clustering patterns and stability trends. A significance threshold of 0.05 was applied to *p*-values across all statistical analyses. To account for multiple testing, *p*-values were adjusted using the Benjamini-Hochberg method implemented by the *p.adjust* function. These adjusted *p*-values, termed *q*-values, were subjected to a significance threshold of 0.1. These corrections accounted for the total number of metabolites assessed in each comparison.

## Results and discussion

This study aimed to assess the feasibility of VAMS in signaling lipid profiling using a previously published plasma-based method that allows the simultaneous extraction of oxylipins, lysophospholipids, free fatty acids, bile acids, endocannabinoids, and a steroid hormone in a single protocol. Analytical performance of VAMS was evaluated in comparison to traditional DBS and WB in terms of precision, LLE recovery, and matrix effect. Endogenous levels were compared across the three matrices to identify potential systematic biases arising from differences in extraction efficiency between the polymeric tip and the filter paper, as well as variations between dried and liquid matrices. Additionally, the short-term stability of signaling lipids in VAMS was evaluated at room temperature across varying storage durations.

### Data overview, quality, reproducibility, and reliability

A total of 182 signaling lipid metabolites were targeted using the two chromatographic methods, including 79 oxylipins, 64 lysophospholipids, 15 fatty acids, 8 bile acids, 15 endocannabinoids, and 1 steroid hormone. The complete list of detected metabolites (*n* = 130), including their common name, systematic name, and compound classification, is provided in Table S1. The distribution and overlap of detected metabolites across sample types and compound classes is summarized in Fig. S1. Lysophospholipids, bile acids, endocannabinoids, and steroid hormone showed the highest and most consistent detection rates across all five sample types. Fatty acids were detected to a lesser extent (~ 50% in WB, VAMS24, and VAMS48; ~ 80% in DBS and VAMS1w), while oxylipins had the lowest detection (~ 27% in WB, VAMS24, and VAMS48; ~ 45% in DBS and VAMS1w). The limited detection of oxylipins likely results from their inherently low concentrations (sub-nanomolar) in blood, often falling below the limit of detection, coupled with the structural complexity arising from numerous regioisomers. These challenges demand high chromatographic resolution and ultra-sensitive mass spectrometry to improve oxylipin detection [37]. The elevated detection of fatty acids and oxylipins in DBS and VAMS1w compared to other sample types suggests potential influences of drying and stability, which will be further explored in the following sections.

**Fig. 2:**
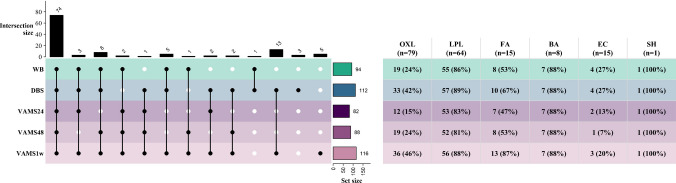
Distribution and intersection of metabolites included in the study across different sample types and compound classes. The figure presents an upset plot illustrating the distribution and overlap of metabolites that passed quality control (background < 40%; precision < 30%), across different sampling methodologies and storage durations. The horizontal bar charts on the right display the total number of metabolites in each sample type. The main matrix represents the intersection of metabolites among sample types, where black circles indicate presence in a given sample type, and vertical bars denote the number of metabolites within each intersection, sorted in descending order. The table provides a class-specific summary, comparing the number of metabolites included in the study to the total number of targets in the two chromatographic methods (*n*). This integrated visualization offers a comprehensive overview of reliable analytical coverage achieved for each compound class, highlighting the influence of sampling methodology and duration of storage at room temperature. BA, bile acids; DBS, dried blood spot; EC, endocannabinoids; FA, fatty acids; LPL, lysophospholipids; OXL, oxylipins; SH, steroid hormone; VAMS, volumetric absorptive microsampling; VAMS24, VAMS stored at room temperature for 24h; VAMS24, VAMS stored at room temperature for 48h; VAMS24, VAMS stored at room temperature for 1 week; WB, whole blood

Four samples (1 DBS post-spike, 1 WB post-spike, 1 VAMS24, and 1 VAMS48) were identified as outliers based on visual inspection of the PCA plot and excluded from further analysis. The precision of ISTDs across the three matrices was within the acceptable range (CV ≤ 30%) for 37 of the 38 measured, except for d3-SEA in WB (Fig. S2). Precision was highest in VAMS, with 31 ISTDs displaying < 15% CV. Notably, VAMS outperformed DBS in 29 ISTDs, demonstrating superior reproducibility. The highest variation was observed in WB, likely due to the inhomogeneity of liquid blood introduced during aliquoting or increased matrix interference impacting LLE in the liquid matrix compared to dried matrices [38]. Of the 130 detected metabolites, 120 met the quality control criteria (background signal < 40% and precision < 30%) and were included in the downstream analyses. A detailed overview of background and precision metrics of the metabolites is provided in Table S2. The distribution and overlap of the metabolites included in this study across sample types and compound classes is illustrated in Fig. 2. Of note, several endocannabinoids were removed due to excessively high background signal, a well-documented issue attributed to interference from solvents and laboratory equipment [22, 39]. Similarly, oxylipins in VAMS24, particularly HDoHEs, exhibited CV > 30%, likely due to regioisomeric co-elution leading to variability in peak integration.

### Analytical performance evaluation with extraction recovery and matrix effect

**Fig. 3:**
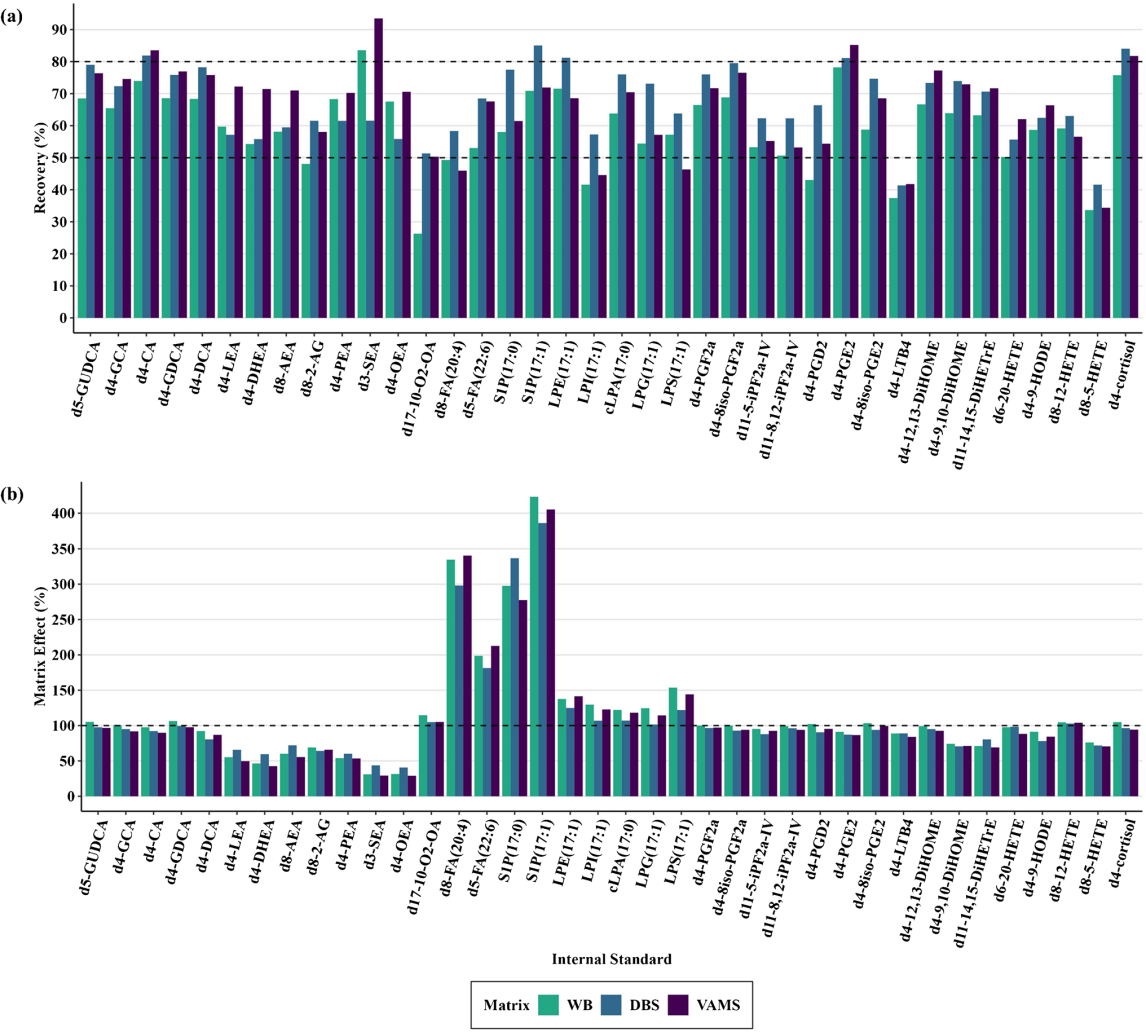
Comparative evaluation of analytical performance across WB, DBS, and VAMS. The figure presents bar plots depicting key parameters of analytical performance across different matrices, based on ISTDs. (**a**) Liquid-liquid extraction recovery across sample matrices, with dashed lines indicating the range within which the majority of the ISTD recoveries fall. (**b**) Matrix effect, with a dashed line at 100%, representing the baseline where no matrix effect is observed. Values above this line indicate ion enhancement, while values below signify ion suppression. DBS, dried blood spot; ISTD, internal standard; VAMS, volumetric absorptive microsampling; WB, whole blood

Extraction recoveries across matrices and lipid classes are illustrated in Fig. 3a and summarized in Table S3. Since ISTDs in DBS and VAMS were added post-sample drying, the observed recoveries strictly reflect the LLE efficiency and do not represent potential variations in analyte extraction from the filter paper or polymeric tip. Most ISTDs exhibited recoveries between 50 and 80%, with DBS yielding the highest recoveries for the majority (*n* = 23). The recovery efficiencies of DBS and VAMS were largely comparable across most compound classes. However, lysophospholipids exhibited greater recovery in DBS, whereas endocannabinoids demonstrated notably higher recoveries in VAMS. This observation suggests that the presence of filter paper or polymeric tip may influence extraction efficiency, potentially through differential analyte interactions with the sampling substrates or co-extraction of interfering compounds specific to each substrate. In contrast, WB consistently exhibited the lowest recoveries in most ISTDs (*n* = 30), indicating a greater interference of enzymes, proteins, and cellular components in liquid matrix than dried matrices during extraction. The drying process in DBS and VAMS likely attenuates these effects by inactivating enzymes, denaturing proteins, and disrupting cellular integrity, thereby enhancing extraction efficiency. Among the lipid classes, bile acids and the steroid hormone showed the highest recoveries across DBS and VAMS, whereas d8-5-HETE and d4-LTB4 from oxylipins exhibited recoveries below 50%, reflecting potential analyte-specific extraction challenges and limitations. To further enhance recoveries, a two-step extraction approach may be implemented to facilitate increased analyte partitioning into the organic phase.

Matrix effects of the different sampling matrices across lipid classes are illustrated in Fig. 3b and summarized in Table S3. By comparing post-spike ISTD in extracted samples to ISTD in pure solvent, this matrix effect analysis eliminates extraction-dependent variations in ISTD recovery, allowing a direct assessment of the impact of co-extracted matrix components on ionization efficiency. Matrix effects varied in both magnitude and direction depending on the compound class, with most exhibiting minimal variation across matrix types. Bile acids and the steroid hormone exhibited the lowest matrix effects, ranging from 81 to 106% and 94 to 105%, respectively, with ion enhancement observed exclusively in WB (where 100% indicates no matrix effect). Similarly, oxylipins displayed matrix effects in the range of 69–105%, with pronounced ion suppression observed in d8-5-HETE (71–76%), d4-9,10-DiHOME (71–74%), and d11-14,15-DiHETrE (69–80%). Endocannabinoids demonstrated the highest susceptibility to ion suppression, ranging from 29 to 72% across matrices, with the greatest effect observed in VAMS. In contrast, lysophospholipids and fatty acids displayed substantial ion enhancement across all matrices, reflecting strong matrix-induced signal amplification. Lysoglycerophospholipids and the oxy-fatty acid exhibited moderate ion enhancement (101–154%), while sphingolipids (278–423%) and free fatty acids (181–340%) displayed extremely high matrix effects. The significant matrix effects observed in endocannabinoids, lysophospholipids, and fatty acids may be attributed to the co-extraction of endogenous phospholipids present in blood samples during LLE, which co-elute with analytes modulating ionization efficiency. Phospholipid-mediated ion suppression and enhancement have been well documented in electrospray ionization-based LC-MS analyses, where they either compete for charge, reducing analyte signal, or facilitate charge transfer, amplifying signal intensity [40–42]. Advanced sample preparation techniques, such as Hybrid-SPE precipitation, have been shown to effectively deplete phospholipids, thereby mitigating matrix effects and improving precision and reproducibility in LC-MS analyses [43]. Another potential contributor to the observed ion enhancement could be the increased adsorption of ISTDs onto the plastic surface of the Eppendorf in the absence of matrix components. Lipophilic compounds have been shown to be highly susceptible to non-specific binding onto polypropylene or polyethylene materials, resulting in significant loss of signal intensity [44, 45]. In the presence of biological matrices, endogenous constituents may passivate the container surfaces, thereby minimizing adsorption and enhancing analyte recovery. Consequently, the reduced signal in neat samples could artificially inflate the apparent analyte response in matrix-containing samples, mimicking ion enhancement.

### Comparison of endogenous metabolite levels across WB, DBS, and VAMS

**Fig. 4:**
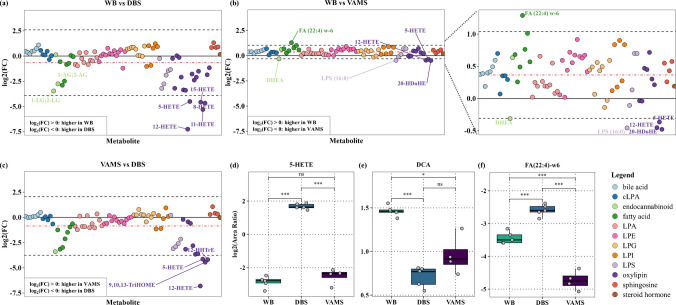
Concordance of endogenous metabolite measurements across WB, DBS, and VAMS. Modified Bland-Altman plots (**a**–**c**) and boxplots (**d**–**f**) compare endogenous metabolite levels across WB, DBS, and VAMS. The modified Bland-Altman plots illustrate the agreement between metabolite measurements across sampling methods by plotting individual metabolites on the *x*-axis and the difference between measurements, expressed as log_2_(FC), on the *y*-axis for (**a**) WB vs DBS, (**b**) WB vs VAMS, and (**c**) VAMS vs DBS. The red dot–dash line denotes the mean difference (systematic bias) between the two methods, while the black dashed lines indicate the upper and lower limits of agreement, defined as the mean difference ± the standard deviation of the differences. The boxplots display the log_2_(area ratio) for an oxylipin (**d**) 5-HETE, bile acid (**e**) DCA, and fatty acid (**f**) FA(22:4)-w6, in each matrix, illustrating trends observed in the Bland-Altman analysis. Each box represents the IQR, with the median indicated by a horizontal line. The whiskers extend to 1.5 times the IQR from the box edges, or to the minimum/maximum value within that range. Individual data points represent the replicate measurements. Statistical significance was determined using *t*-tests and displayed, with *p*-values denoted as “ns” (*p* > 0.05), “*” (*p* ≤ 0.05), “**” (*p* ≤ 0.01), and “***” (*p* ≤ 0.001). DBS, dried blood spot; FC, fold change; IQR, interquartile range; VAMS, volumetric absorptive microsampling; WB, whole blood

The agreement between WB, DBS, and VAMS for endogenous metabolite measurements was assessed using modified Bland-Altman plots, evaluating systematic biases, variability, and methodological differences. Figure 4a–c illustrates the observed trends across different compound classes in the pairwise comparisons of WB-DBS, WB-VAMS, and VAMS-DBS, elucidating the impact of sampling techniques on metabolite stability and matrix-dependent extraction efficiency. A systematic discrepancy was evident between DBS and WB, with DBS consistently yielding higher signals across most metabolites, as indicated by the negative mean bias in Fig. 4a. The broad limits of agreement (LOA), spanning −3.9 to 2.6 log_2_ fold change, further underscore the substantial variability between these sampling methods. The most pronounced differences were observed in oxylipins, lysophosphatidylserines (LPS), and acylglycerol endocannabinoids, suggesting potential influences of pre-analytical factors such as non-enzymatic oxidation, degradation, or differences in extraction efficiency between matrices. Among the metabolites, HETEs were identified as extreme outliers, falling outside the LOA and exhibiting significantly higher levels in DBS compared to WB (Fig. 4d). As HETEs are established autoxidation products of arachidonic acid, their substantial increase in DBS demonstrates that prolonged drying at room temperature can introduce oxidative artifacts [46]. Similarly, the higher levels of acylglycerols in DBS may stem from differences in extraction efficiency, as evidenced by the d8-2-AG ISTD, which showed lower LLE recovery in WB (48%) compared to DBS (62%) (Table S3), suggesting that poor analyte recovery in WB may contribute to the observed discrepancies. Further, although K2 EDTA serves as an effective anticoagulant by chelating calcium ions, it does not completely inhibit platelet activation, which can be triggered by mechanical stress or exposure to ambient conditions. Upon activation, platelets have been shown to release bioactive lipids, including 12-HETE through the lipoxygenase pathway and 2-AG via the diacylglycerol lipase activity [47, 48]. Therefore, the elevated levels of HETEs and 2-AG observed in DBS may also reflect potential platelet-mediated lipid release induced during the drying process, thereby contributing to the discrepancies between DBS and WB metabolite profiles.

In contrast to the WB-DBS comparison, WB-VAMS revealed a small positive mean bias, indicating that most metabolites were detected at marginally higher levels in WB than in VAMS (Fig. 4b). Notably, the LOAs were considerably narrower, spanning a log_2_ fold change of −0.3 to 1, demonstrating that VAMS aligned more closely with WB than DBS. Although HETEs exceeded the lower LOA, their increase in VAMS was minimal as demonstrated by the absence of statistically significant differences (Fig. 4d). The extent of autoxidation was significantly lower in VAMS than DBS, as shown by the substantially higher levels of HETEs in DBS (Fig. 4c), reinforcing the hypothesis that endogenous metabolites are more susceptible to modifications on DBS. A study by Petrick et al. demonstrated that while both DBS and VAMS remained stable at −80 °C and −20 °C storage, VAMS exhibited superior stability at 4 °C for metabolomic analysis [49]. While our study assessed short-term stability at room temperature, the greater resilience of VAMS under suboptimal storage conditions further supports its potential advantages for metabolomic analyses where immediate frozen storage is not feasible. These trends suggest a potential protective effect in VAMS, likely attributed to the 3D porous design of its polymeric tip, which facilitates rapid and uniform drying, reduces enzymatic degradation and platelet activation, and minimizes surface area exposure to environment, thereby increasing metabolite stability. However, the consistent lower levels observed in VAMS compared to WB also highlight the possibility of reduced extraction efficiency from the polymeric tip, potentially suggesting stronger analyte retention within the sampling substrate. This demonstrates the need for further optimization of the extraction protocol to enhance analyte recovery from VAMS while maintaining its advantages in mitigating oxidative degradation. The most pronounced differences in WB-VAMS were observed in bile acids, fatty acids, LPEs, and LPGs, with higher levels observed in WB. A similar pattern was observed in WB-DBS, where bile acids, LPEs, and LPGs were also higher in WB compared to DBS, implying minor degradation of these lipid classes in dried matrices after a storage of 24h at room temperature (Fig. 4e). Furthermore, these compounds were closely centered around the zero line in the VAMS-DBS comparison (Fig. 4c), reinforcing the hypothesis that their reduced levels in dried matrices likely stem from degradation during storage rather than matrix-dependent variability. Alternatively, fatty acids exhibited an opposing trend, with higher levels in DBS and lower levels in VAMS relative to WB (Fig. 4f), indicating that fatty acid extraction from the polymeric tip may be less efficient with the current sample preparation. This observation may be attributed to the non-polar nature of fatty acids, which could result in stronger interactions with the hydrophobic VAMS tip, thereby limiting their complete recovery during extraction.

### Short-term stability of lipids in VAMS at room temperature

**Fig. 5:**
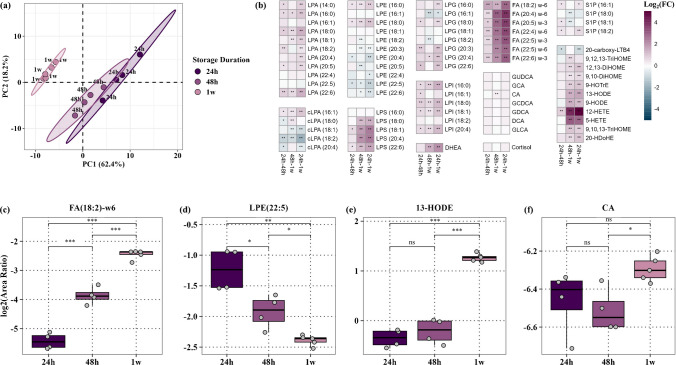
Stability of metabolites in VAMS over 24h, 48h, and 1 week storage at room temperature. (**a**) PCA visualizes the clustering of samples based on storage duration, with each point representing a replicate measurement and distinct clustering patterns indicating temporal shifts in metabolite profiles. (**b**) The heatmap depicts the log_2_(FC) in metabolite levels between time points (24h–48h, 48h–1 week, and 24h–1 week), with color scale representing the magnitude and direction of change and statistical significance marked as “*” before and “**” after multiple testing correction. The boxplots illustrate the log_2_(area ratio) of (**c**) FA(18:2)-w6, (**d**) LPE(22:5), (**e**) 13-HODE, and (**f**) CA, across the three time points, highlighting trends in metabolite stability. Each box represents the IQR with median indicated by a horizontal line. The whiskers extend to 1.5 times the IQR from the box edges, or to the minimum/maximum value within that range. Individual data points represent the replicate measurements. Statistical significance was determined using *t*-tests and displayed, with *p*-values denoted as “ns” (*p* > 0.05), “*” (*p* ≤ 0.05), “**” (*p* ≤ 0.01), and “***” (*p* ≤ 0.001). DBS, dried blood spot; FC, fold change; IQR, interquartile range; PCA, principal component analysis; VAMS, volumetric absorptive microsampling; WB, whole blood; 24h, 24 h storage at room temperature; 48h, 48 h storage at room temperature; 1w, 1 week storage at room temperature

The stability of signaling lipids in VAMS was assessed to examine its practical feasibility for storage at room temperature. The PCA plot revealed distinct clustering of samples based on the duration of storage (Fig. 5a). Notably, VAMS1w exhibited the greatest separation, primarily along the first principal component (PC1) which accounted for 62% of the total variance, indicating that extended storage for 1 week at room temperature significantly alters the sample composition. In contrast, VAMS24 and VAMS48 were differentiated along PC2, which explained 18% of the variance, suggesting more subtle yet detectable metabolic changes from 24 to 48 h. Temporal variations in endogenous metabolite profiles were visualized using heatmaps depicting fold changes across VAMS24 vs VAMS48, VAMS48 vs VAMS1w, and VAMS24 vs VAMS1w, with statistical significance identifying metabolites prone to oxidative and hydrolytic degradation (Fig. 5b). Of the 77 metabolites detected across all three storage time points, 60 metabolites changed significantly (*q* < 0.05) over time (Table S4). The most consistent and significant increases from VAMS24 to VAMS48 to VAMS1w were observed in free fatty acids (Fig. 5c). This accumulation may be attributed to the residual lipase activity or hydrolytic degradation of complex lipids in the sample, leading to the release of free fatty acids over time [50]. The concomitant decline of several LPEs and cLPAs further supports this hypothesis, suggesting that their breakdown may contribute to the observed elevation of fatty acids (Fig. 5d). Additionally, prolonged storage may induce matrix alterations, which could potentially alter the extraction efficiency of analytes from the polymeric tip. Other lysophospholipids, including LPAs, LPIs, LPGs, and LPSs, predominantly exhibited increasing trends over time, with variations in the magnitude of change. LPS species showed the most significant increase at 1 week, with minimal or no change between 24 and 48h, suggesting a delayed accumulation effect. In contrast, LPAs, LPIs, and LPGs displayed more gradual and subtle increases, with some species remaining completely stable.

A similar pattern was observed for oxylipins, which remained relatively stable in the early storage period, with no significant differences between VAMS24 and VAMS48, except in 12-HETE which increased significantly, which may be attributed to minor platelet activation during wicking. Pronounced increases were detected in VAMS1w samples, suggesting that the initial stabilization of oxylipins is time limited (Fig. 5e). The significant rise in HETEs, HODEs, DiHOMEs, and TriHOMEs, well-established lipid peroxidation products of polyunsaturated fatty acids, indicates that prolonged storage at room temperature facilitates non-enzymatic autoxidation. In contrast, bile acids and the steroid hormone demonstrated the highest stability, with no significant differences across storage durations. The only exception was cholic acid, which increased significantly from 48h to 1 week, potentially reflecting the degradation of its conjugated counterparts under prolonged storage, suggesting that conjugated species may be susceptible to hydrolysis over extended storage conditions (Fig. 5f). Overall, these findings highlight the variable stability of lipid classes in VAMS and emphasize the need for optimized storage strategies, particularly for oxidation- and hydrolysis-prone lipids. To minimize these metabolomic alterations, VAMS samples should be immediately stored at low temperatures post-drying, ideally at −80 °C, to preserve lipid integrity. However, since VAMS are widely utilized for at-home sampling, delays in sample transport to the laboratory may prolong exposure to ambient conditions. Storage of samples with desiccants could mitigate hydrolytic degradation by reducing moisture, thereby enhancing the stability of lipids. Furthermore, pre-treating VAMS tips with antioxidants, such as butylated hydroxytoluene, may offer protection against oxidative degradation [51]. However, the concentration of antioxidants utilized could influence the absorptive capacity of VAMS, necessitating extensive validation to ensure optimal preservation without compromising volumetric accuracy of sampling.

## Conclusion

While VAMS has emerged as a promising microsampling technique for metabolomics, its potential in signaling lipid profiling remains largely unexplored. This study assessed the feasibility of VAMS for the comprehensive profiling of diverse signaling lipid classes using a single extraction protocol, with its analytical performance benchmarked against traditional matrices of WB and DBS. Our findings establish VAMS as a suitable sampling methodology for signaling lipid analysis, demonstrating its ability to detect oxylipins, lysophospholipids, fatty acids, bile acids, endocannabinoids, and steroid hormones, with superior precision to DBS. While LLE recovery was moderate, it remained comparable to DBS, suggesting that optimization of sample preparation could improve analyte extraction. Notably, the sampling substrates appeared to influence LLE recovery for lysophospholipids and endocannabinoids, suggesting potential interactions between these lipid species and the filter paper in DBS or the polymeric tip in VAMS. Furthermore, dried matrices demonstrated greater extraction efficiency than the liquid matrix, indicating that the drying process may mitigate matrix interferences, thereby enhancing analyte recovery. Matrix effects were mostly similar across WB, DBS, and VAMS, indicating that the sampling substrate itself does not significantly influence ionization efficiency. A pivotal finding of this study is that VAMS retained a metabolic profile more closely aligned with WB than DBS, suggesting better lipid preservation during the critical 24h drying phase at room temperature. This enhanced stability indicates that VAMS mitigates oxidative and hydrolytic degradation as well as platelet activation more effectively than DBS, reinforcing its potential for use in remote or decentralized sampling where immediate cold storage is not feasible.

In addition to these advantages, the study also identifies areas for further improvement and future research. While the use of a single healthy volunteer allowed a proof-of-concept evaluation of the feasibility of VAMS for signaling lipid profiling without introducing biological variability, it limits the generalizability of the findings. Future research must incorporate a larger cohort to validate and strengthen the reliability of the results. The limited detection of oxylipins highlights the need for more sensitive mass spectrometry platforms to capture low-abundance lipid mediators. As lipid recovery may be influenced by sampling substrate interactions, future studies should investigate extraction efficiency from the filter paper and polymeric tip to quantify the proportion of original metabolite levels successfully recovered and assess whether tailored protocols can improve extraction performance. This can be achieved by spiking known concentrations of standards into blood prior to sampling to quantify unrecovered fractions. Further, to isolate losses attributable to the substrate alone, comparison of recovery from samples spiked before collection with those spiked after collection will help distinguish substrate-specific retention from losses incurred during subsequent processing steps, such as LLE. The short-term stability assessment provided valuable insights into the impact of room-temperature storage on signaling lipid stability over 1 week. However, many real-world applications, such as home-collection and postal shipments, may involve longer durations and variable temperatures, which could further affect lipid stability. Therefore, future studies should explore lipid stability under extended and fluctuating storage conditions. Additionally, strategies to minimize degradation, such as cold storage, desiccant-assisted storage, or antioxidant pretreatment, should be further investigated. While this study evaluated a minimum storage period of 24h, future research should investigate the extent of metabolic changes during the early drying and storage phases by comparing samples stored for shorter durations (e.g., 2, 4, and 6h) and at different temperatures to better understand the kinetics of lipid degradation under initial storage conditions.

In conclusion, this exploratory study demonstrates that VAMS is a viable and robust alternative for comprehensive signaling lipid profiling, offering distinct advantages over traditional DBS. By combining volumetric accuracy, high precision, and enhanced stability, VAMS bridges the gap between decentralized sampling and analytical reliability. While further research in the optimization of extraction protocols and stabilization strategies is needed, this study lays a strong foundation for its integration in clinical applications, biomarker research, and precision medicine.

## Supplementary Information

Below is the link to the electronic supplementary material.

Supplementary file1 (DOCX 289 KB)

Supplementary file2 (XLSX 67.6 KB)

## Data Availability

All data supporting the findings of this study are available within the paper and its Electronic Supporting Material.

## Abbreviations

ACN: Acetonitrile
BuOH: Butanol
CV: Coefficient of variation
DBS: Dried blood spots
ISTD: Internal standard
LC-MS: Liquid-chromatography mass-spectrometry
LLE: Liquid-liquid extraction
LOA: Limits of agreement
LPS: Lysophosphatidylserine
MeOH: Methanol
MTBE: Methyl *tert*-butyl ether
PC: Principal component
UHPLC-MS/MS: Ultra-high-performance liquid-chromatography tandem mass-spectrometry
VAMS: Volumetric absorptive microsampling
WB: Whole blood
