# Supplementary material for: Volumetric absorptive microsampling for profiling of signaling lipids: a comparative analysis with whole blood and dried blood spots
Source: Anal Bioanal Chem. 2026 Apr 5;418(9):2565–78. doi: 10.1007/s00216-026-06413-5 (PMC13079519; doi:10.1007/s00216-026-06413-5)
Supplement: Supplementary file 1 — Supplementary file1 (DOCX 289 KB) [file 216_2026_6413_MOESM1_ESM.docx]

Title: Volumetric Absorptive Microsampling for Profiling of Signaling Lipids: A Comparative Analysis with Whole Blood and Dried Blood Spots

Journal Title: Analytical and Bioanalytical Chemistry

Authors: Manchu Umarani Thangavelu^1^, Alida Kindt^1^, Bert Wouters^1^, Lieke Lamont^1^, Hyung Lim Elfrink^1^, Amy Harms^1^, *Thomas Hankemeier^1^

Affiliations: ^1^Metabolomics and Analytics Center, Leiden University, Leiden, The Netherlands.

*Corresponding Author Email: [hankemeier@lacdr.leidenuniv.nl](mailto:hankemeier@lacdr.leidenuniv.nl)


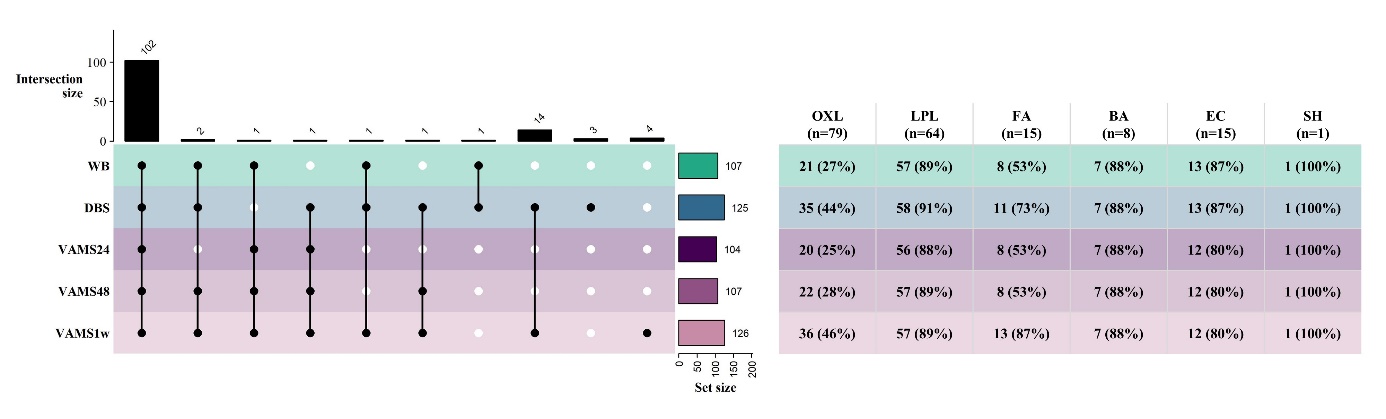


**Fig. S1. Distribution and intersection of detected metabolites across different sample types and compound classes.** The figure presents an upset plot illustrating the distribution and overlap of metabolites detected across different sampling methodologies and storage durations. The horizontal bar charts on the right display the total number of metabolites in each sample type. The main matrix represents the intersection of metabolites among sample types, where black circles indicate presence in a given sample type, and vertical bars denote the number of metabolites within each intersection, sorted in descending order. The table provides a class-specific summary, comparing the number of metabolites detected to the total number of targets in the two chromatographic methods (n). This integrated visualization offers a comprehensive overview of analytical coverage achieved for each compound class, highlighting the influence of sampling methodology and duration of storage at room temperature. BA: bile acids; DBS: dried blood spot; EC: endocannabinoids; FA: fatty acids; LPL: lysophospholipids; OXL: oxylipins; SH: steroid hormone; VAMS: volumetric absorptive microsampling; VAMS24: VAMS stored at room temperature for 24 hours; VAMS24: VAMS stored at room temperature for 48 hours; VAMS24: VAMS stored at room temperature for 1 week; WB: whole blood.


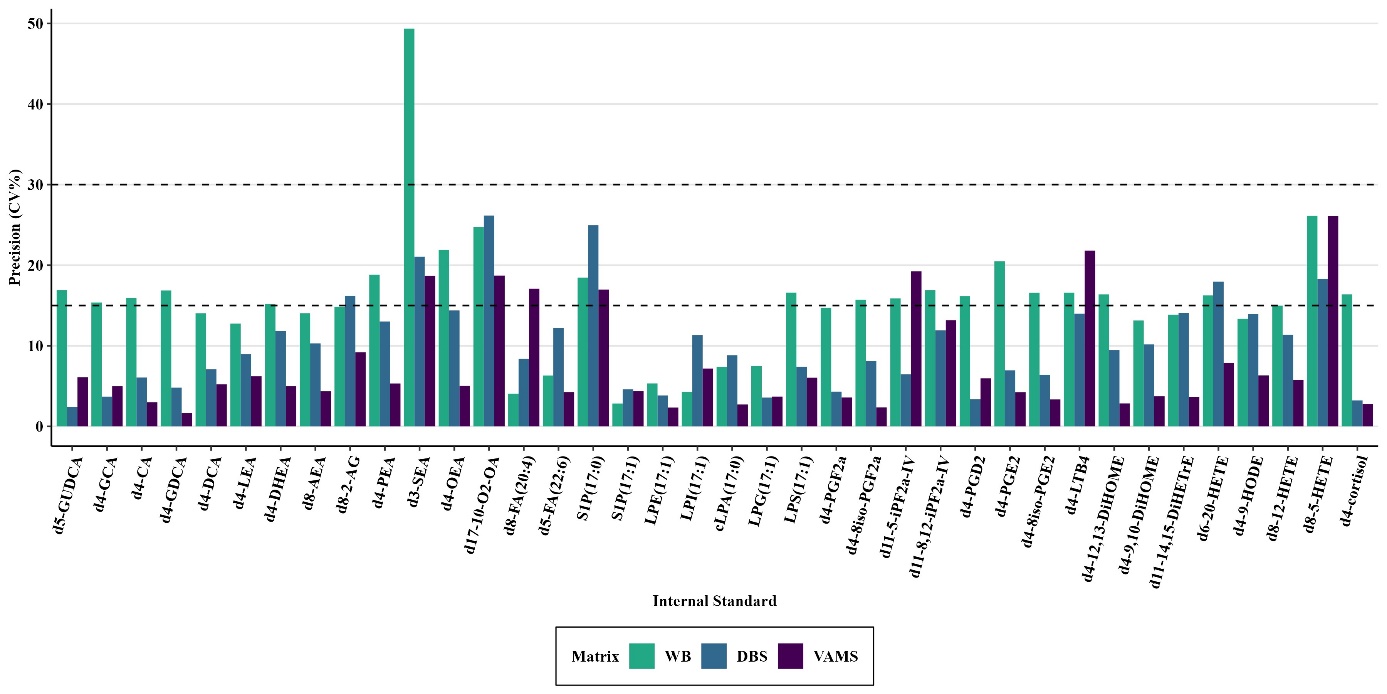


**Fig. S2. Precision of internal standards across WB, DBS, and VAMS matrices.** The figure presents a bar plot depicting the precision of 38 internal standards across WB, DBS, and VAMS. Precision is expressed as the coefficient of variation (%), with dashed lines indicating commonly applied acceptance thresholds. This visualization provides a comparative assessment of reproducibility across sampling methodologies, highlighting differences in analytical variability between liquid and dried matrices. CV: coefficient of variation; DBS: dried blood spot; VAMS: volumetric absorptive microsampling; WB: whole blood.
